# Noninvasive Brain Stimulation and Psychotherapy in Anxiety and Depressive Disorders: A Viewpoint

**DOI:** 10.3390/brainsci9040082

**Published:** 2019-04-14

**Authors:** Moussa A. Chalah, Samar S. Ayache

**Affiliations:** 1 Service de Physiologie, Explorations Fonctionnelles, Hôpital Henri-Mondor, AP-HP, 94010 Créteil, France; samarayache@gmail.com; 2 EA 4391, Excitabilité Nerveuse et Thérapeutique, Université Paris-Est-Créteil, 94010 Créteil, France; 3 Neurology Division, Lebanese American University Medical Center-Rizk Hospital (LAUMC-RH), Beirut 1100, Lebanon

**Keywords:** transcranial direct current stimulation, tDCS, transcranial magnetic stimulation, TMS, intermittent theta burst stimulation, cognitive behavioral therapy

## Abstract

Among the most prevalent psychiatric conditions stand anxiety and depression. Psychotherapy and medications are considered effective treatments in these clinical settings. However, pharmacotherapy and psychotherapy (i.e., cognitive behavioral therapy (CBT)) administered in monotherapy or in a combined regimen do not result in satisfactory outcomes in all patients. Therefore, finding new treatments would be of great help. In the last three decades, noninvasive brain stimulation (NIBS) has emerged as a safe tool to improve several neuropsychiatric symptoms. The following work revisits the available reports that assessed the add-on value of NIBS techniques when combined to psychotherapy (CBT or related interventions) in mood and anxiety disorders. The available protocols targeted the prefrontal cortex, a region that was previously found to have an enhanced activity or functional connectivity after psychotherapeutic interventions. Promising yet scarce evidence exists on this matter. A discrepancy exists among the available reports regarding the type and duration of interventions, the patients’ clinical profiles, and the presence of a sham intervention. NIBS may have acted by enhancing psychotherapy effects on the top-down cognitive control of emotions. Combining both therapies may result in promising effects, but future large-scale trials are needed to judge the utility of this combination in psychiatric populations.

## 1. Introduction

Among the most prevalent psychiatric conditions stand anxiety and depression. The lifetime prevalence of anxiety disorders and major depressive disorder (MDD) are respectively 25%–30% and 17% [1,2]. Both are characterized by a young age of onset (adolescence or young adulthood), reduced quality of life, and altered social and professional functioning [3]. A cognitive model has been proposed by Beck to account for the occurrence of these symptoms and constitutes the basis of psychotherapy (i.e., cognitive therapy) developed in this field [3]. In this model, anxiety and depression may arise from a selective heightening of bottom-up processes at the level of emotionally ‘hot’ areas (e.g., hippocampus, amygdala) that are involved in generating fear-related (in the case of anxiety) or negative (in the case of depression) thoughts; or an aberrant (top-down) cognitive control of emotions which occurs at the level of emotionally ‘cold’ cortical areas (e.g., prefrontal cortex (PFC)) [3]. 

Psychotherapy, such as cognitive behavioral therapy (CBT), is considered an effective treatment for these conditions [3]. CBT involves changing maladaptive behaviors by allowing patients’ to experience the consequence of the latter (i.e., behavioral therapies), modifying false beliefs or cognitions (e.g., cognitive therapies), and combining several strategies (e.g., mindfulness therapy, cognitive diffusion, acceptance of unwanted thoughts) in an attempt to improve the thinking process and modulate the effects of symptoms. CBT may act by enhancing the top-down cognitive control of emotions and/or reducing the bottom-up effects of the latter [3]. 

However, only 50%–65% of patients with anxiety disorders exhibit significant clinical changes following exposure-based CBT, which is considered a first line treatment in this population [4]. The same applies to MDD since pharmacotherapy and psychotherapy (i.e., CBT) administered in monotherapy or combined could only result in partial remission or no improvement, as reported in up to 30 % of patients [5,6]. 

Therefore, finding new treatments would be of great help in boosting the outcomes of the currently available interventions. In the last three decades, noninvasive brain stimulation (NIBS) has emerged as a safe tool to improve several neuropsychiatric symptoms [7,8]. NIBS consists of applying a magnetic field (the case of repetitive transcranial magnetic stimulation (rTMS)) or a weak electrical current (the case of transcranial direct current stimulation (tDCS)) over the scalp area overlying the cerebral target. 

rTMS is based on the electromagnetic induction law introduced by Michael Faraday in 1831 [7]. According to this law, delivering a time-varying current through a coil placed over the scalp would induce a magnetic field that could induce in turn a secondary electrical current in a neighboring conducting milieu such as the human cortical networks [7]. The magnetic pulses could be delivered with different frequencies. High frequency (≥ 5Hz) and low frequency (≤1 Hz) rTMS seem to exert excitatory and inhibitory effects on cortical excitability, respectively [7]. This widely accepted consensus is based on neurophysiological studies in healthy subjects where the application of high-frequency and low-frequency rTMS over the primary motor cortex resulted in an increase and a decrease in the amplitude of motor evoked potentials (MEPs), respectively [7]. 

In addition to the classical rTMS paradigms, new ones have been developed, such as theta burst stimulation (TBS). As its name implies, TBS mimics endogenous theta rhythms, and consists of delivering large number of pulses in a relatively short duration. TBS can be applied in an intermittent (iTBS) or a continuous (cTBS) manner. The former is believed to induce excitatory effects and the latter has been found to have inhibitory impact on cortical networks. Again, this dichotomy is based on the effects exerted by TBS on MEPs amplitude [7]. 

As for tDCS, it consists of delivering a weak electrical current (i.e., 1–2 mA) via two saline-soaked sponge electrodes (i.e., anode and cathode) connected to a battery-driven stimulator over a period of 10–30 min [8]. Anodal stimulation seems to depolarize neuronal membrane, thus exerting excitatory effects on the neural circuits [8]. Opposite effects were seen with cathodal stimulation. Similar to rTMS and TBS, the previously described effects of tDCS are based on studies that assessed MEPs changes following the application of tDCS over the motor cortex. 

It is worth mentioning that, for all these techniques, the dichotomy (excitatory vs. inhibitory) was also adapted when attempting to inhibit or activate non-motor cortical regions such as the prefrontal cortex [7]. However, it is important to highlight that aside from the stimulation type, other variables could dictate the physiological outcome and the effects seen in the motor cortex may not apply to other cortical sites. 

It is also important to note that the observed effects, whether inhibitory or excitatory, result from the capacity of NIBS to induce neuroplasticity processes (long-term potentiation and long-term depression) and to modulate the activity of the stimulated area and its functional connectivity with other cerebral regions [9,10,11]. 

Both techniques (tDCS and rTMS) have been recommended in the treatment of psychiatric disorders. The actual evidence supports definite antidepressant effects of rTMS (Level A of evidence) when applied over the left dorsolateral PFC and possible anxiolytic effects (Level C) when applied over the right dorsolateral PFC (i.e., in patients with post-traumatic stress disorder (PTSD)) [7]. As for tDCS, probable efficacy (Level B) has been documented in patients with MDD when anodal stimulation is applied over the left dorsolateral PFC [8]. 

The safety profile of these techniques renders them appealing to adapt in clinical practice. They might also serve as add-on therapies to psychotherapy in psychiatric patients. The current work revisits the available literature that combined psychotherapy (i.e., CBT or its components such as exposure therapy admitting its role in anxiety and related disorders) with NIBS (i.e., tDCS, rTMS and related interventions) in patients with psychiatric disorders. A particular focus is given to depressive disorders, bipolar disorders, and anxiety disorders as classified in the diagnostic and statistical manual for mental disorders (DSM), fifth edition [12]. In addition, two other categories (i.e., obsessive compulsive and related disorders, and trauma- and stressor-related disorders) were considered, since they were previously part of anxiety disorders in the fourth edition of DSM [13]. Admitting the scarcity of the available data, the current work considered all types of reports published at any time on this matter until February 2019 (i.e., sham-controlled trials, open-label studies and case reports). 

## 2. Noninvasive Brain Stimulation and Anxiety Disorders

### 2.1. Panic Disorder and Agoraphobia

In line with the cognitive model of anxiety, patients with panic disorders were found in some neuroimaging studies to have an alteration in the ‘fear network’ with a hypoactivity reported in PFC which takes part in inhibiting fear-related emotions via its links with subcortical structures (e.g., amygdala) [14]. The first insight on combining CBT and NIBS in this context derives from a randomized sham-controlled trial [14] that recruited patients with panic disorder who received 9 weeks of group psychotherapy (9 CBT sessions including exposure therapy sessions that help facilitating fear extinction [15]). During CBT, patients underwent 15 sessions of active or sham left iTBS applied over the left dorsolateral PFC (F3 according to the 10–20 electroencephalogram (EEG) system of electrode positioning; for details on stimulation parameters please review [16]). In addition to clinical assessment, patients were evaluated using functional near-infrared spectroscopy during the performance of an emotional (Stroop) task before and after the treatment protocols, and their imaging data were compared to a group of healthy controls. At baseline, compared to healthy controls, patients exhibited reduced left prefrontal activation in response to panic-related stimuli (compared to neutral stimuli (words)). Only active iTBS resulted in bilateral prefrontal activation. However, both stimulation arms did not differ in clinical outcomes except on agoraphobic avoidance at 6-month follow-up after CBT, which was more stably reduced in the active treatment arm. Active iTBS may have served to maintain CBT effects over time. However, the dissociation between the clinical and imaging data at the end of treatment deserves to be further addressed in order to assess the utility of NIBS as an add-on therapy for psychotherapy. Interestingly, in another randomized sham-controlled study by the same authors, 15 daily sessions of active or sham iTBS over the left dorsolateral PFC (F3 according to 10–20 EEG system) were applied over three weeks, combined with a total of 3 weekly group sessions of psychoeducation [17]. Active iTBS did not appear to augment psychoeducation effects in the considered patients suffering from panic disorder/agoraphobia nor did it result in enhancing the frontal hypoactivation pattern documented at baseline. Compared to the first study, which included 9 CBT sessions, 3 sessions of psychotherapy in the second study may not have been sufficient to induce changes similar to those observed in the first study. 

### 2.2. Phobia of Heights or Acrophobia

In a recent randomized sham-controlled trial, acrophobic patients underwent two treatment sessions each comprising a virtual reality exposure therapy applied following high-frequency rTMS (session duration: 20 min, intensity: 100% resting motor threshold (rMT), frequency: 10 Hz, *n* = 1560 pulses/session) over the ventromedial PFC (Fpz according to 10–20 EEG system), a region that has a key role in fear extinction learning based on clinical and experimental studies [4]. This protocol was based on a previous work that has documented an rTMS-induced increase in ventromedial PFC activity and better extinction learning in healthy controls [18]. Following the combined treatment, anxiety and avoidance ratings were significantly better in the active rTMS group, supporting the relevance of rTMS as an add-on therapy for exposure intervention. However, at 3-month follow-up, acrophobia symptoms further improved to an equal level in both arms. In light of these findings, active rTMS may have acted by accelerating the onset of psychotherapy effects. 

### 2.3. Spider Phobia or Arachnophobia

Two randomized sham-controlled studies considered patients with spider phobia and applied left dorsolateral prefrontal iTBS (a single active or sham session over F3 according to 10–20 EEG system) followed by a virtual reality challenge. In the first study, groups were compared during the performance of the same emotional (Stroop) task using functional near-infrared spectroscopy [19]. At baseline, patients exhibited left inferior frontal gyrus hypoactivation in response to emotionally irrelevant words compared to healthy controls. However, such difference did not remain at the end of the protocol, highlighting the positive effects of exposure therapy, but challenging the add-on value of iTBS, since neither of the stimulation conditions yielded additional benefits. In the second study, the authors reported the psychophysiological effects of the previous protocol [20]. Similarly, a single session of active or sham iTBS did not affect heart rate or skin conductance, both of which increased following treatment compared to baseline, independent of stimulation type. However, it is worth noting that active iTBS was able to modulate heart rate variability. In both works, the absence of additional effects following active iTBS might be due to a ceiling effect of virtual reality or insufficient number of sessions (1 session).

### 2.4. Obsessive Compulsive Disorder

The literature on obsessive compulsive disorder (OCD) derives from a case report and an open label study. In the first work, the authors enrolled an adult woman suffering from treatment-resistant OCD that did not previously respond to serotonergic antidepressants, atypical antipsychotics, or 16 CBT sessions [21]. During her acute presentation, the patient did not respond to 12 weeks of citalopram infusion, but clinically improved after receiving 16 CBT sessions, 10 of which were combined with high frequency rTMS over the left dorsolateral PFC (intensity: 80% rMT, frequency: 10 HZ, *n* = 1800 pulses/session). The patient’s clinical improvement was still persistent two years later, and was accompanied by an improvement in quality of life and global level of functioning. The failure of the previous CBT trial when administered alone suggests that rTMS was able to prime CBT effects in this patient, despite the fact that placebo effects related to rTMS cannot be ruled out here. These findings were replicated in an open-label study involving 18 patients with treatment-resistant OCD admitted for severe loss of functioning [22]. Patients were all treated with a combination of pharmacotherapy, CBT, and rTMS over the left dorsolateral PFC (intensity: up to 110% rMT, frequency: 25 Hz, *n* = 1000 pulses/session). Such a combination resulted in significant improvement of symptoms, but the conclusion is challenged by the open-label nature of the study, and the difference in the adapted number of CBT or rTMS session among patients (mean number of rTMS sessions: 23.28 ± 6.78; mean number of CBT sessions: 17.17 ± 5.04). A third double-blind study has applied exposure therapy followed by 25 rTMS sessions over the medial PFC and anterior cingulate cortex (ACC), regions that seem to be hyperactivated in the context of OCD [23]. Better clinical results were obtained in the high frequency arm (intensity: 100% leg rMT, frequency: 20 Hz, *n* = 2000 pulses/session) compared to the low frequency (intensity: 110% leg rMT, frequency: 1 Hz, *n* = 900 pulses/session) and sham stimulation arms, a finding that was accompanied by physiological (EEG) changes in the ACC activity. Here, it is worth noting that the lack of significant effects with low frequency stimulation might be related to the lower number of pulses applied in this condition rather than to the low frequency per se. 

### 2.5. Post-Traumatic Stress Disorder

PTSD trials focused on combining NIBS with exposure therapy. In a sham-controlled and cross-over trial, subjects suffering from chronic and treatment-refractory PTSD received 20 sessions of active or sham low-frequency rTMS over the right dorsolateral PFC (session duration: 30 min, intensity: 100% rMT, frequency: 1 Hz, *n* = 1800 pulses/session) with imaginal exposure therapy [24]. The choice of stimulation target derives from a positron emission tomography study where patients with PTSD were found to have an increased activity in this region during symptom provocation [25]. Larger, yet statistically non-significant effect size of improvement in hyperarousal symptoms was obtained following active compared to sham rTMS. Similarly, in a recent randomized sham-controlled parallel trial, patients with PTSD randomly received exposure therapy combined with 5 weekly high-frequency right or left dorsolateral prefrontal rTMS sessions (rTMS session duration: 30 min, intensity: 120% motor threshold, frequency: 10 Hz, *n* = 6000 pulses/session) [26]. A nonsignificant trend toward improvement was obtained regarding PTSD symptoms, and significant antidepressant effects were obtained in patients with comorbid depression. In a third sham-controlled study, 30 patients randomly received 12 sessions of high frequency rTMS applied over the medial PFC after the exposure to traumatic or non-traumatic imagery (session duration: 15.5 min, intensity: 120% rMT, frequency: 20 Hz, *n* = 1680 pulses/session) [27]. Compared to the control groups (active rTMS following exposure to non-traumatic imagery or sham rTMS following exposure to traumatic imagery), a significant reduction in PTSD symptoms was observed with real rTMS following exposure to traumatic imagery. Compared to the first two studies, the significant results obtained in the third one might be related to the relatively larger sample size (*n* = 9 [24] and *n* = 8 [26] vs. *n* = 30 [27]), the difference in the cerebral targets (dorsolateral PFC [24,26] vs. medial PFC [27]), the stimulation parameters (low frequency in [24] and high frequency in [26,27]) and the clinical characteristics of the recruited cohorts. 

## 3. Noninvasive Brain Stimulation and Depression 

The available NIBS reports were designed based on the prefrontal imbalance hypothesis which implies a hypoactive left dorsolateral PFC and a hyperactive right dorsolateral PFC in patients with MDD. This model is based on functional neuroimaging and neurophysiological studies [28,29,30]. Therefore, activating the left side or inhibiting the right side using NIBS techniques has been previously proposed and tested [7,8]. 

Regarding rTMS, the first report concerns a 26-year-old woman with treatment-resistant MDD who was treated over 14 weeks with 39 sessions of high frequency rTMS targeting the left dorsolateral PFC, of which 14 were combined with CBT (each session: 30 min in duration, intensity: 120% rMT, frequency: 10Hz, *n* = 6000 pulses/session) [31]. The patient gradually improved following treatment and remained in remission for at least three months afterwards. In a large recent naturalistic study, at least 10 sessions of cognitive behavioral therapy combined with rTMS (10 Hz over the left dorsolateral PFC, 1500 pulses/session or 1 Hz over the right dorsolateral PFC, 1200 pulses/session or both kinds of stimulations sequentially) in 196 patients with MDD [32]. This combination resulted in 66% response rate and 56% remission rate at the end of the therapy. At six months, sustained remission reached 60%. Although the data are promising, the lack of sham control merits to be further addressed in future works.

As for tDCS, one report concerned a 52-year-old woman with severe and chronic MDD that did not response for multiple drugs and two individual psychotherapies (CBT and psychodynamic psychotherapy) [33]. Following 10 daily sessions of bifrontal tDCS applied over two weeks (anode and cathode: F3 and F4 respectively according to the 10–20 EEG system, current intensity: 1.5 mA), the patient experienced a remarkable improvement that partially disappeared in the 4-week follow-up period. Afterwards, the authors repeated tDCS treatment combined with weekly CBT sessions (performed after the electric current start) that lasted six months. The patient was in remission until at least the one-year follow-up, despite the end of the protocol and the reduction of pharmacological therapy. Given that the patient did not previously respond to psychotherapies, including CBT, and taking into consideration the previously observed vanishing tDCS effects, both techniques may have complemented each other’s. On the one hand, CBT could have prolonged tDCS effects on depressive symptoms. On the other hand, tDCS may have primed CBT response in a previously resistant patient. Such synergistic effects should be interpreted with caution again due to the absence of a sham stimulation. 

In a recent randomized double-blind sham-controlled trial [34], 14 patients with MDD received four weeks of computer-based CBT (12 modules) combined with 12 sessions of active or sham bifrontal tDCS (three days per week for four weeks; anode over F3 and cathode over F4, 10–20 EEG system). In each tDCS session, a 2 mA current was applied over 30 minutes. All patients improved compared to their baseline scores regardless of the intervention, but the authors report that the number of patients who completed the protocol was too small to be able to perform a statistical group comparison (active versus sham) and judge the add-on value of tDCS when combined with CBT. 

To overcome this limitation, a recent ongoing randomized sham-controlled multicenter trial is assessing the benefits of combining left prefrontal tDCS with CBT in patients with MDD [35]. 192 patients will receive 6-week of group CBT alone (12 sessions each lasting 60 min), group CBT combined to sham tDCS, or group CBT combined with active bifrontal tDCS performed during psychotherapy sessions (anode over F3 and cathode over F4, 10–20 EEG system, current intensity: 1–2 mA, applied 10 min after starting CBT and lasting 30 min). The prefrontal activity and connectivity will be evaluated before and after interventions using functional MRI. The results of this study are highly awaited.

## 4. Current Knowledge and Future Perspectives

The few available data consisted of fourteen reports, of which nine were sham-controlled trials, two were open-label studies, and three were case reports. To start, regarding panic disorder/agoraphobia, the only sham-controlled trial combined CBT with 15 iTBS sessions targeting the left PFC and did not yield prominent clinical benefits [14]. In the context of acrophobia, the available sham-controlled trial suggests the role of high-frequency prefrontal rTMS in accelerating treatment response to exposure therapy [4]. In patients with spider phobias, two sham-controlled trials employing exposure therapy failed to document an add-on value of left prefrontal iTBS, probably because a single stimulation session is insufficient to induce effects [19,20]. OCD studies have combined NIBS with CBT (in a case study [21] and an open label protocol [22]) and exposure therapy (in a sham-controlled trial) and yielded positive effects that are limited by the lack of sham arm [21,22] or the scarcity of data [21,22,23], warranting replication in future large-scale studies. The available sham-controlled trials on PTSD have combined rTMS with exposure therapy, and resulted in statistically significant effects in one study [24], marginal effects in a second study [26], and no effects in a third one [27]. Finally, in the context of MDD, positive data were obtained following the application of left prefrontal rTMS in one case report and one open label study, and after left prefrontal anodal tDCS in one case report [31,32,33]. However, in the only available randomized controlled trial [34], the add-on value of tDCS could not be studied because of the small sample size. 

A discrepancy exists among the available studies regarding the type of psychotherapy intervention, the number of psychotherapy and NIBS sessions, the pharmacological profiles of treated patients, the presence of psychiatric comorbidities and the presence of a sham intervention. All the reported studies applied NIBS techniques over the PFC, a region that was previously found to have an enhanced activity or an increase in its functional connectivity with other areas (fronto-limbic connections) following psychotherapy [3,36]. Therefore, tDCS and rTMS may have acted by enhancing CBT effects on the top-down cognitive control of emotion. Although combining both therapies may result in promising effects, it is premature to draw formal conclusions based on the present findings, and future large-scale randomized trials are needed in order to judge the utility of this combination in psychiatric populations. 
